# De-implementation strategy to reduce unnecessary antibiotic prescriptions for ambulatory HIV-infected patients with upper respiratory tract infections in Mozambique: a study protocol of a cluster randomized controlled trial

**DOI:** 10.1186/s13012-024-01382-8

**Published:** 2024-07-16

**Authors:** Candido Faiela, Troy D. Moon, Mohsin Sidat, Esperança Sevene

**Affiliations:** 1 Department of Biological Science, Faculty of Science, Eduardo Mondlane University, Maputo, Mozambique; 2 Department of Tropical Medicine and Infectious Diseases, School of Public Health and Tropical Medicine, Tulane University, New Orleans, USA; 3 Department of Community Health, Faculty of Medicine, Eduardo Mondlane University, Maputo, Mozambique; 4 Department of Physiological Science, Faculty of Medicine, Eduardo Mondlane University, Maputo, Mozambique

**Keywords:** Antimicrobial stewardship, Acute respiratory infections, Protocol, Implementation science, De-implementation, Clinical decision support tool, HIV, Mozambique

## Abstract

**Background:**

Antibiotics are globally overprescribed for the treatment of upper respiratory tract infections (URTI), especially in persons living with HIV. However, most URTIs are caused by viruses, and antibiotics are not indicated. De-implementation is perceived as an important area of research that can lead to reductions in unnecessary, wasteful, or harmful practices, such as excessive or inappropriate antibiotic use for URTI, through the employment of evidence-based interventions to reduce these practices. Research into strategies that lead to successful de-implementation of unnecessary antibiotic prescriptions within the primary health care setting is limited in Mozambique. In this study, we propose a protocol designed to evaluate the use of a clinical decision support algorithm (CDSA) for promoting the de-implementation of unnecessary antibiotic prescriptions for URTI among ambulatory HIV-infected adult patients in primary healthcare settings.

**Methods:**

This study is a multicenter, two-arm, cluster randomized controlled trial, involving six primary health care facilities in Maputo and Matola municipalities in Mozambique, guided by an innovative implementation science framework, the Dynamic Adaption Process. In total, 380 HIV-infected patients with URTI symptoms will be enrolled, with 190 patients assigned to both the intervention and control arms. For intervention sites, the CDSAs will be posted on either the exam room wall or on the clinician´s exam room desk for ease of reference during clinical visits. Our sample size is powered to detect a reduction in antibiotic use by 15%. We will evaluate the effectiveness and implementation outcomes and examine the effect of multi-level (sites and patients) factors in promoting the de-implementation of unnecessary antibiotic prescriptions. The effectiveness and implementation of our antibiotic de-implementation strategy are the primary outcomes, whereas the clinical endpoints are the secondary outcomes.

**Discussion:**

This research will provide evidence on the effectiveness of the use of the CDSA in promoting the de-implementation of unnecessary antibiotic prescribing in treating acute URTI, among ambulatory HIV-infected patients. Findings will bring evidence for the need to scale up strategies for the de-implementation of unnecessary antibiotic prescription practices in additional healthcare sites within the country.

**Trial registration:**

ISRCTN, ISRCTN88272350. Registered 16 May 2024, https://www.isrctn.com/ISRCTN88272350

**Supplementary Information:**

The online version contains supplementary material available at 10.1186/s13012-024-01382-8.

Contribution to the literature

- Antibiotic use for acute upper respiratory tract infections is usually unnecessary, as most infections are viral and self-limited. Interventions that promote the de-implementation of this practice will contribute in turn to combat the global threat of antimicrobial resistance.
- This multicenter cluster randomized controlled trial assesses if a multiprong de-implementation strategy anchored on a clinical decision support algorithm will promote practice change and thus reduce unnecessary antibiotic prescriptions.
- De-implementation has been understudied in primary healthcare settings in low- and middle-income countries. This study will add to the evidence base around how to de-implement unnecessary antibiotic use in a primary healthcare setting.

## Background

Upper respiratory tract infections (URTIs) are common in adults worldwide [1]. These infections are typically diagnosed clinically based on predominant signs and symptoms and then classified according to their anatomical location such as nasopharyngitis, pharyngitis, tonsillitis, or otitis media [2]. Approximately 90% of all URTIs are of viral etiology and the use of antibiotics may not be indicated [3]. Despite this, as much as 50% to 70% of patients with URTI end up being prescribed antibiotics [4]. Excessive or inappropriate antibiotic use for URTIs is considered a low-value and unnecessary practice and thus needs to be de-implemented [5]. De-implementation is perceived as an important area of research that can lead to reductions in unnecessary, wasteful, or harmful practices [6].

Strategies to promote the de-implementation of unnecessary and wasteful antibiotic use should focus not only on appropriate use but also on the sustainability of behavioral change for both clinicians and patients [7]. Improving antibiotic prescribing requires complementary strategies which include changing clinician behavior and educating patients and families about the role of antibiotics in medical care and their well-being. Commitment to these strategies by clinicians and other relevant health workers may optimize antibiotic prescribing and patient safety. Several studies have recommended appointing a clinical “champion” to promote appropriate antibiotic prescribing [7, 8]. Clinicians who have demonstrated ownership of the process are more likely to be committed to the appropriate use of antibiotics [7].

In high-income countries, several strategies are being employed to promote the de-implementation of unnecessary antibiotic use in patients with URTI across a variety of different clinical settings. These strategies include 1) the use of clinical decision support algorithms (CDSA) by antibiotic prescribers; 2) employment of rapid diagnostic testing or a biomarker to try and reduce uncertainty in diagnosis in real-time, and thus the need for empiric antibiotics; 3) education of healthcare providers, including feedback and auditing concerning their prescribing practices; 4) establishing institutional antibiotic stewardship programs; and 5) creation of, and then deployment of essential medicines policies [9–19].

CDSAs are effective among several strategies to promote the de-implementation of inappropriate prescribing, mainly when combined with the education of health workers [20]. CDSAs have been used in both printed form and within electronic prescribing systems. CDSAs developed for the management of respiratory tract infections have shown significant implementation effectiveness [7]. A considerable number of these tools have been integrated into electronic prescribing platforms and have been associated with reduced inappropriate antibiotic prescribing [7, 11, 21]. In situations in which an electronic platform may not be available, such as in many low- and middle-income countries (LMIC) there is evidence documenting the successful implementation of CDSA in printed form. Rambaud-Althaus et al. evaluated the effect of either a print version of a CDSA or a smartphone-based electronic version and compared it with a control group in a primary healthcare setting. The authors found a significant reduction in antibiotic use in both the printed paper (26%) and electronic CDSA arms (25%), as compared to the control arm (70%) [11].

In persons living with HIV (PLHIV), URTIs are the main reason for an antibiotic being prescribed, especially in those patients with a low viral load [3, 22]. With advances in antiretroviral therapy (ART), the risk of URTIs in PLHIV has reduced over time and is now similar to HIV-uninfected individuals [23]. One exception to this is the HIV-infected patients who quit taking their ART, increasing their vulnerability and risk of getting an infection [24].

In Mozambique, the approach for treating URTIs among HIV-infected patients in the outpatient setting is predominantly empirical and often results in the prescription of antibiotics despite strong etiologic evidence of a bacterial infection. Our previous work found a high frequency of antibiotic prescriptions being given to HIV-infected patients (65.9%) in the outpatient settings, mostly for respiratory tract infections, and recommended the development of strategies to promote the reduction of unnecessary antibiotic use in this population [25]. At the time, it was felt that one potential solution to this problem would be the utilization of a CDSA that could help clinicians differentiate when a patient with acute respiratory symptoms needs antibiotics versus those who do not, thus ideally reducing the number of unnecessary antibiotics being prescribed [6, 26]. In this research, we hypothesize that the CDSA, when added to the usual or routine care, is effective as part of a de-implementation strategy in reducing unnecessary antibiotic prescriptions.

Interventions that promote the de-implementation of unnecessary, wasteful, or harmful practices may improve the quality of patient care and reduce the empirical use of antibiotics [6]. Considering that PLHIV are subject to taking medications their entire life, reducing the use of antibiotics, will contribute to reducing the number of medications these patients are exposed to taking and therefore reducing the likelihood of drug interactions and adverse reactions [27].

We summarize below our protocol for this de-implementation study, describing the conceptual frameworks that have guided its development, the different phases that will be employed throughout its implementation, and the measures we propose for evaluating its implementation and effectiveness.

## Methods/Design

### Aims and objectives

The overall aim of this study is to evaluate the implementation and effectiveness of a de-implementation strategy for reducing the unnecessary use of antibiotics for the treatment of acute URTIs in HIV-infected adult patients that are being managed in select ambulatory primary healthcare clinics (PHC) in Mozambique. The focus of this evaluation is a multifaceted de-implementation strategy that includes a combination of interventions including health worker education, audit and feedback, organizational adjustments, and the introduction of a CDSA for decision-making around antibiotic use. We subsequently evaluate its implementation and effectiveness as a function of the RE-AIM conceptual framework. The reporting of this protocol adheres to the SPIRIT checklist [28].

### Study setting

This study will be implemented within outpatient primary healthcare clinics in Maputo (the nation’s capital city) and Matola (a city approximately 30 min outside Maputo which is the capital of Maputo Province) municipalities in southern Mozambique. Both municipalities are subdivided into 10 administrative units, hereafter referred to as clusters, containing a total of 31 eligible primary healthcare facilities. All healthcare facilities providing primary care to HIV-infected patients within the study area will be eligible and enlisted for the randomization process.

### Study design and conceptual frameworks

**Fig. 1:**
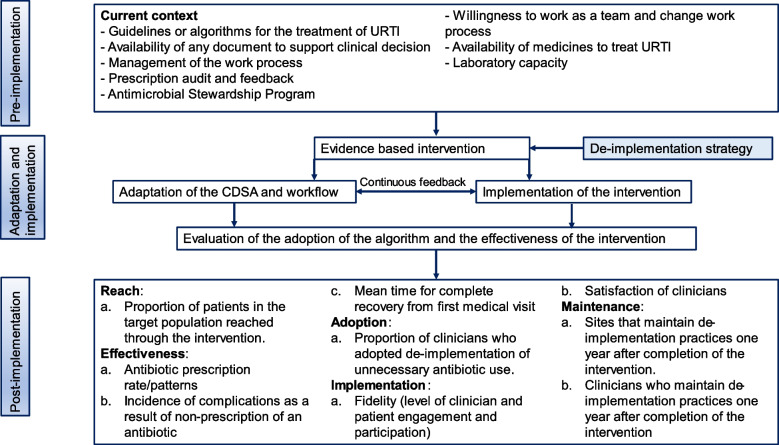
The framework of the dynamic adaptation process of the intervention

We propose a multicenter two-arm cluster randomized controlled trial that will employ a mixed-methods approach. This study will be guided by two conceptual frameworks, the Dynamic Adaptation Process (DAP) and the Reach, Effectiveness, Adoption, Implementation, and Maintenance (RE-AIM) framework, and will be carried out in three phases (Fig. 1). The DAP framework was developed to provide the structure for an iterative process to guide, monitor, and evaluate the introduction of a new intervention into practice. DAP is a framework that allows changes to be made according to the real context by the possibility of tailoring the elements of the intervention based on data obtained during the pre-implementation and the adaptation. DAP engages stakeholders at all levels to develop robust implementation strategies and will guide the work of phases one, two, and three in our study [29]. *Phase one* (pre-implementation) will consist of a formative baseline evaluation of the current situation for antibiotic prescribing for URTI in the outpatient setting among PLHIV and occur over three months. It will consist of interviews with identified health workers to understand the current antibiotic prescribing practices for URTI among HIV-infected ambulatory patients, as well as perceived facilitators and barriers to the roll-out of our de-implementation strategy. A document review will be performed to identify the existence of national guidelines or normative documents driving antibiotic prescribing for URTI. Phase one will also include documentation of current laboratory diagnostic capacity for URTI and a situational assessment of the availability of medicines to treat URTI. *Phase two* (adaptation and implementation) consists of our de-implementation strategy roll-out and will occur over six months. Based on information learned in phase one, we will make workflow adjustments to address realities on the ground. The CDSA will be rolled out within a two-arm cluster randomized control trial design in which our primary outcome measure will be the clinical decision to use antibiotics or not. Intervention elements and study tools will be adapted before the intervention is implemented. Throughout this phase, an implementation audit and continuous feedback will be conducted to guarantee and monitor adherence to the intervention protocol. *Phase three* (post-implementation) will consist of a three-month post-implementation phase in which we will analyze implementation outcomes and processes as a function of the RE-AIM conceptual framework, in real-time. *Reach* will be assessed in terms of patient recruitment, refusal, and attrition. *Effectiveness* will be analyzed by comparing antibiotic use rates and patient-related clinical outcomes between the intervention and control arms. For *adoption*, clinicians who adopted the practice of de-implementation of unnecessary antibiotic use will be assessed using prescriptions and clinical records. For *implementation*, the fidelity and satisfaction of clinicians regarding the intervention will be assessed. For *maintenance*, sites and clinicians that maintain de-implementation practices and use of the CDSA will be assessed one year after completion of the intervention.

### Randomization within the two-arm cluster randomized trial and characteristics of the study participants

To mitigate contamination threat to internal validity among participating facilities, randomization and allocation will be primarily among the 10 administrative units (primary clusters). Primary clusters are considered municipal districts (Maputo) or administrative posts (Matola). Randomization will be performed before the initiation of our pre-implementation phase. Initially, randomization will be undertaken through the generation of a sequence of random numbers corresponding to each primary cluster. A total of six primary clusters will be randomly assigned to either the intervention or control arms (three each). Afterwards, in each selected primary cluster only one primary healthcare facility (secondary cluster) will be randomly selected to participate in the study. All participants in the same facility will be assigned to the same treatment, either intervention or control. A statistician will generate the allocation sequence and assign participating healthcare facilities to intervention or control groups.

**Fig. 2:**
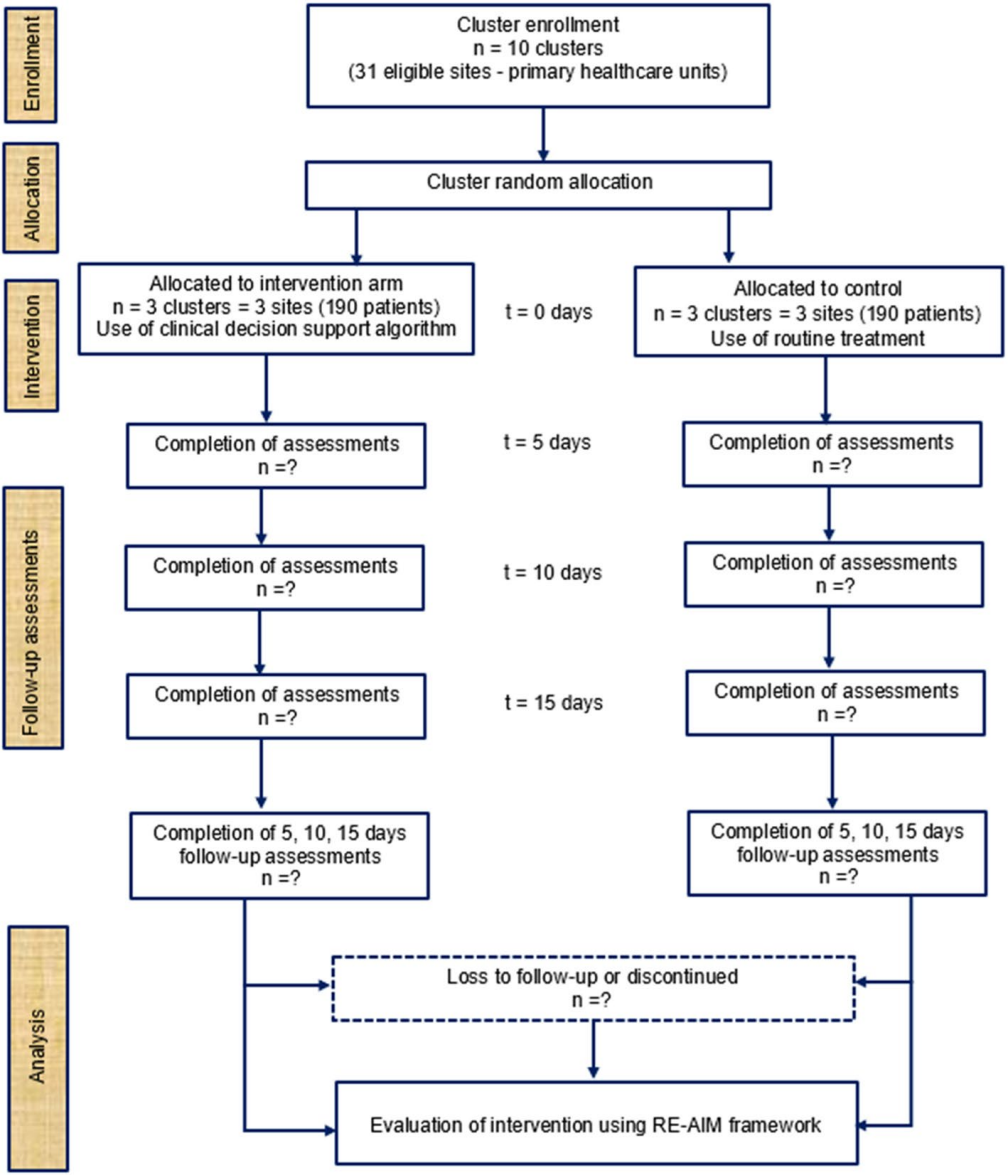
Study flow diagram: enrollment, intervention, and assessments

During phase one (pre-implementation), we will enroll 42 health workers as part of our formative baseline assessment. Eligible health workers will include clinicians, laboratory technicians, pharmacists, and health managers at each of our six study facilities. During phase two (adaptation and implementation), all clinicians at each site working with adult HIV-infected patients aged 18 years and older will be enrolled in the study. Eligible health workers are nurses, clinical officers (called *Técnicos de Medicina* in Portuguese), and physicians. Health workers attending to pediatric patients and those not engaged in the HIV outpatient clinic will be excluded from the study. One identified person (health worker or manager) from each cluster facility will function as a local coordinator, responsible for coordinating activities on site and being the contact between healthcare providers participating in the intervention and the research team. A sample of 380 HIV-infected patients with URTI will be enrolled and assigned to each study arm in a 1-to-1 ratio (190 for each arm) (Fig. 2). To achieve adequate participant enrollment, all clinicians who examine HIV-infected patients in the screening and outpatient consultation rooms at each selected health facility will be requested to identify potential participants among their patients, and the study team will invite them to participate in the study.

For phase three (post-implementation), we will re-interview all clinicians from each of the intervention sites concerning aspects of the intervention implementation and satisfaction.

### Intervention and control

Eligible patients allocated to intervention will be managed using the CDSA. Eligible patients are adult HIV-infected with respiratory symptoms (nasal secretions or runny nose, congestion, sore throat, coughing, sneezing, chills, smell and taste disorders, with or without a fever) [30]. Adult HIV-infected patients without URTI symptoms, those with fever ≥ 39ºC, severe mental illness, or advanced HIV status will be excluded from the study. All PLHIV with URTI symptoms lasting less than 10 days will not receive an antibiotic unless there is an additional symptom suggesting a suspect of bacterial infection. Otherwise, bacterial infection will be suspected in the following situations: (i) higher-grade fever than normally observed with the common cold, with the presence of yellow or greenish nasal discharge, pain/difficulty swallowing, or an intense sore throat; (ii) URTI symptoms lasting longer than 10 days; (iii) URTI symptoms continuing to get worse rather than improve over several days (5 days after the first visit) [31].

**Fig. 3:**
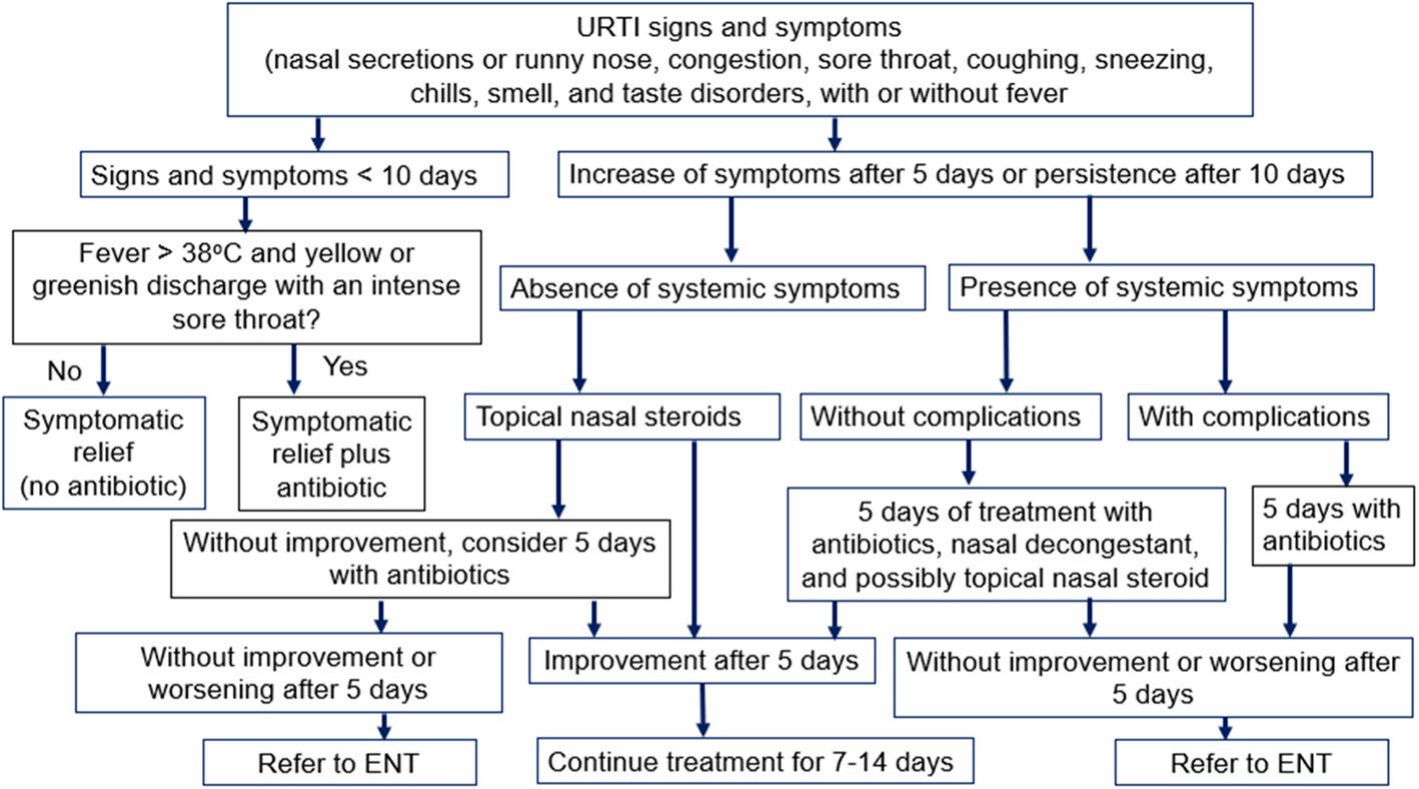
Clinical decision support algorithm

Decongestants and/or antihistamines may be used to relieve symptoms of cough, congestion, and runny nose at the clinician's discretion [32]. Those with an increase in symptoms after five days, or a persistence of symptoms after 10 days, without systemic symptoms, will be treated with topical nasal steroids. If improvement of symptoms is observed after five days of treatment with nasal steroids, patients will continue with treatment for seven to fourteen days. Without improvement, consider 5 days of treatment with antibiotics. If improvement, patients should continue with treatment for seven to fourteen days. If symptoms get worse after 5 days of antibiotics, patients will be referred to an ear-nose-throat specialist (ENT). On the other hand, those with systemic symptoms without complications will follow five days of treatment with antibiotics, nasal decongestants, and topical nasal steroids (Fig. 3). Those with complications (acute otitis media, sinusitis, bronchitis, and pneumonia) will be given five days of treatment with antibiotics. Then, without improvement after 5 days of antibiotics, patients will be referred to an ear-nose-throat specialist. The promotion of de-implementation of unnecessary antibiotic use for HIV-infected patients with URTI symptoms lasting less than 10 days will be the core strategy of using the CDSA in the experiment arm.

Patients allocated to control will follow usual or routine treatment and will be used as a comparator group. The control arm will allow a fair comparison of the effectiveness of the intervention. No specific intervention will be assigned to the control arm except for follow-up. The clinicians will decide to prescribe medication during each medical visit as they are used to do. For both arms, patients will be enrolled for the first time (t0 = 0 days) and monitored three times (t1 = 5 days, t2 = 10 days, t3 = 15 days) after the initial medical visit to see improvement of symptoms through a phone call, and if necessary, will be asked to visit the healthcare facility for a follow-up clinical examination in person (Fig. 2). If the patient fails to answer the calls at t1, t2, or t3, a home visit on the following day will be made for a follow-up procedure. Participation will be discontinued if the patient’s HIV clinical condition worsens as a result or not of the intervention and appropriate care and treatment will be provided including hospitalization if required.

### De-implementation strategy

The process to tailor the de-implementation of unnecessary antibiotic prescriptions will combine multifaceted interventions which include health worker education, organizational adjustments, audit and feedback, and roll-out of the CDSA (Table 1). We will start the intervention period by holding an educational meeting with clinicians from the three intervention sites about the de-implementation of unnecessary antibiotic prescriptions to treat URTI as indicated in the CDSA. During the meeting, we will explain to the prescribers how to use the CDSA. In this meeting, we will distribute the CDSA to each participant. In addition, CDSAs will be posted on either the exam room wall or on the clinician´s exam room desk for ease of reference during clinical visits. Further, based on the results of our baseline assessment about potential barriers and facilitators to the use of our CDSA, we will work with the facility to make any organizational adjustments that may be necessary to address potential barriers. In each participating facility, we will identify one local coordinator who will function as a local champion for this intervention, be responsible for the enrollment of participants and obtaining informed consent from potential trial participants or surrogates, and serve as the study focal point for coordination with the study researchers. Finally, throughout the implementation phase, the study researchers will conduct a prescription audit and feedback on 50% of the antibiotic prescriptions that were made during this period. They will then meet with intervention site clinicians, every other week, to review the audit findings and provide feedback as to maximizing the use of the CDSA.

**Table 1:** Summary of de-implementation strategy

Item	Description
Education	Educate clinicians in using the CDSA highlighting the de-implementation of unnecessary antibiotic prescriptions to treat URTI
Organizational adjustments	We will adjust the workflow based on the local setting to fit the intervention
Audit and feedback	Some prescriptions will be audited in the clinical sessions and feedback will be given. Once in two weeks, the principal investigator will meet the local coordinator or champion to monitor and evaluate the records and feedback will be given to improve the implementation of the intervention
Roll-out the CDSA	A4 poster with the algorithm to support clinical decisions will be posted on the wall of the consultation room or placed on the table

### Data collection and measures

- Phase one (Pre-implementation)

Data for the study will be drawn from multiple sources. For phase one, we will collect qualitative data about the current context of both intervention and control sites regarding the treatment of URTI among outpatient HIV-infected patients. We will conduct in-depth interviews among relevant healthcare workers which will include clinicians, pharmacists, and laboratory technicians using interview forms to guide and collect data. Interviews will include a combination of close-ended and open-ended questionnaires to explore current antibiotics prescribing practices for URTI, current workflow of patients through the facility, management of work processes, existence of current practices and normative documents in performing prescription audits and feedback, existence of any initiatives for implementing an antimicrobial stewardship program (ASP), willingness to work as a team to change work processes, availability of medicines to treat URTI, and current laboratory capacity for diagnosing the etiology or a URTI.

- Phase two (adaptation and implementation) and phase three (post-implementation)

Throughout phases two and three we will collect both quantitative and qualitative data. For each HIV-infected patient determined to be eligible for this study, we will collect information related to their socio-demographics, current symptoms, and management decisions related to antibiotics. If antibiotics are prescribed, we will collect data on the type of antibiotic, and expected length of treatment. Antibiotic prescriptions data will be collected from both pharmacy and medical records. To assess whether a clinician has adopted the de-implementation of unnecessary antibiotic use, the duration of symptoms and prescriptions will be reviewed in the clinical records. This will be measured as the proportion of clinicians who adopted the de-implementation of unnecessary antibiotic use practices among those who participated in the study. The “Reach” outcome will be measured as the proportion of patients in the target population reached by the intervention (in terms of # patients recruited, # patients refused and # patients recruited and dropped out during the intervention). This data will be collected from the study data record form.

Qualitative data will be collected to measure the satisfaction of health workers regarding the implementation of the intervention including information related to adoption and acceptability of the intervention. This data will be collected through in-depth interviews with intervention site clinicians, using an interview guide and will be audio recorded. The interview guide will contain open-ended questions related to the adoption, fidelity, and acceptability of the intervention. An additional survey will be used to assess the degree of clinicians’ satisfaction. Maintenance will be measured as both the number of sites and clinicians that maintain the use of the de-implementation strategy one year after completion of the intervention implementation. This data will be collected from prescriptions and clinical records.

Secondary outcomes consist of the incidence of complications as a result of non-prescription of an antibiotic and the mean time for complete recovery from the first medical visit. This information will be collected from the study data record form. The incidence of complications will be measured as the proportion of complications arising from non-prescription of an antibiotic among patients who did not receive antibiotics. The mean time for complete recovery will be measured as the average time that the patients took to recover completely from their symptoms.

### Data management and statistical analysis

We will design our study forms in REDCap (Research Electronic Data Capture), a secure web data capture tool developed by Vanderbilt University that offers a range of functions to collect, store, and analyze basic data from the desired population. Forms will be stored and de-identified in REDCap with access to all study researchers and the REDCap data manager of the Faculty of Medicine of Eduardo Mondlane University. The forms will be exported to SPSS (Statistical Package for Social Sciences) version 25 for statistical analysis.

- Quantitative data analysis

Descriptive and inferential statistical analysis deemed relevant based on collected data will be performed. The descriptive analysis will be based on the elaboration of absolute and relative frequency tables and charts. To explore the factors associated with antibiotic prescribing and the need for antibiotic prescribing, multivariate logistic regression analysis will be performed. The dependent variables to be considered separately will be the prescription of antibiotics and the need for the antibiotic. Each dependent variable will be crossed with the possible factors (independent variables). ANOVA will be used to test differences among sites within the same group for both intervention and control sites with a significance level of 5%.

To compare the effect of the intervention between the intervention and control sites, Pearson's chi-square test will be used, with a significance level of 5%. To verify the magnitude of the effect of the intervention, the relative risk (RR) will be calculated and to estimate the effectiveness of the intervention, the effectiveness ratio (1 – RR) will be determined. For inferential analysis, the 95% confidence interval will be calculated for the RR parameter.

For power calculation, we have set alpha equal to 0.05 and 80% power to detect differences in proportion greater than or equal to 0.15. In other words, if we get a reduction of 15% of the overall antibiotic rate in the intervention sites compared to control sites, we could detect a significant change in proportion. We assumed a coefficient of variation equal to 0.2 for this estimate.

Quantitatively, satisfaction will be measured as the degree of satisfaction of the prescribing clinicians at the intervention study sites using the CDSA. To identify the factors that influence their degree of satisfaction using the CDSA, a multivariate logistic regression analysis will be performed. For the dependent variable, the satisfaction of clinicians, the 5-point Likert scale will be reduced to a binary category of dissatisfied (very dissatisfied, dissatisfied, neutral) and satisfied (very satisfied, satisfied). Potential factors that influence satisfaction to be considered are sex, age, length of time in current position, and the professional category of the health worker.

- Qualitative data analysis

Qualitative data will be coded and analyzed using qualitative analytic software (*NVivo version 12*). Audio recordings will be transcribed and coded using constructs consistent with our study aims and relevant implementation outcomes (adoption, acceptability) which will be outlined in a codebook with definitions. After encoding the constructs, a directed content analysis approach with allowance for the emergence of new themes will be used. After the qualitative analysis, we will perform a quantitative analysis of the contents or themes using a frequency table to be created in the Excel program.

### Trial status

The trial commenced recruitment in June 2024, and all the sites have already started enrollment.

### Dissemination plans

The results of this study will be discussed with all health professionals who participated in it, with the relevant stakeholders at the provincial level, and with the Ministry of Health. Reports and scientific publications will be used to disseminate the results to the broader scientific community. Results will be disseminated regardless of the magnitude or direction of the effects.

## Discussion

This study will evaluate the effectiveness and implementation of a CDSA in promoting the de-implementation of unnecessary and wasteful antibiotic prescriptions in treating acute URTI among an HIV-infected adult population in an ambulatory clinic setting. Most acute URTIs have a viral etiology and antibiotic use for these conditions is perceived as unnecessary and inappropriate [1]. The de-implementation of unnecessary antibiotic use will result in a reduction of antibiotic prescription rates and in turn, contribute to combat antibiotic resistance. Inappropriate and unnecessary antibiotic use for acute URTI is among the main contributors to the development of antibiotic resistance [22, 23]. Considering that HIV-infected patients take medicines their entire lives, the reduction of antibiotic use will reduce the number of medicines prescribed to them, thus reducing the chance of potential interactions and adverse reactions [33].

Evidence suggests that the use of a CDSA can improve management and reduce antibiotic prescription rates. Tabatabaei et al., evaluated the feasibility of a new CDSA in reducing rates of misdiagnosis and inappropriate use of antibiotics for the treatment of acute respiratory tract infections (ARTI) in pediatric patients [34]. The study concluded that the use of the new CDSA was feasible and could help to reduce diagnostic errors and the frequency of antibiotic prescriptions in pediatric patients with ARTI. Shao et al., in a quasi-experimental study in primary health care, evaluated a CDSA to improve antibiotic use in the integrated management of childhood illness [9]. The study observed a statistically significant improvement in adherence to CDSA use between the control group and the intervention group. The study concluded that using the new CDSA improved clinical outcomes and reduced antibiotic prescribing by 80%. However, the CDSAs used in these two studies are differentiated and different from the CDSA proposed here. The studies described above were carried out in primary healthcare facilities and among pediatric patients. This study will be carried out in primary healthcare settings, but in adult and HIV-infected patients.

The randomized design employed in this study will strengthen our results and help to create a fair comparison across the intervention and control arms. Additionally, the study will be targeting a relatively simple and short-term intervention. Short-term and simple interventions are generally viewed as easier to de-implement than more complex interventions [6].

Findings from this study could be scaled up to additional primary health care settings expanding de-implementation practices. To our knowledge, this will be the first study in Mozambique targeting the de-implementation of unnecessary antibiotic use practices in treating acute URTIs among an ambulatory HIV-infected population. It will contribute to the literature on de-implementation science by determining whether the use of CDSA is an effective strategy for promoting the de-implementation of unnecessary and wasteful antibiotic prescriptions to HIV-infected patients.

### Supplementary Information

Supplementary Material 1.

## Data Availability

The protocol and all data generated will be available from the corresponding authors upon request and will be deposited at the Faculty of Medicine, Eduardo Mondlane University data repository.

## Abbreviations

ART: Antiretroviral therapy
ARTI: Acute respiratory tract infection
ASP: Antimicrobial Stewardship program
CDSA: Clinical Decision Support algorithm
CNBS: National Bioethics Committee for Health (*Comité Nacional de Bioética para Saúde*)
DAP: Dynamic Adaptation Process
ENT: Ear, Nose, and Throat Specialist
HIV: Human immunodeficiency virus
PHC: Primary healthcare
PLHIV: People living with HIV
RE-AIM: Reach, effectiveness, adoption, implementation, and maintenance framework
URTI: Upper respiratory tract infections
